# Correction: Dagenais et al. Real-World Safety of CFTR Modulators in the Treatment of Cystic Fibrosis: A Systematic Review. *J. Clin. Med.* 2021, *10*, 23

**DOI:** 10.3390/jcm11020318

**Published:** 2022-01-10

**Authors:** Renée V. E. Dagenais, Victoria C. Su, Bradley S. Quon

**Affiliations:** 1 Adult Cystic Fibrosis Program, St. Paul’s Hospital, Vancouver, BC V6Z 1Y6, Canada; VSu@providencehealth.bc.ca (V.C.S.); bradley.quon@hli.ubc.ca (B.S.Q.); 2 Department of Pharmacy, St. Paul’s Hospital, Vancouver, BC V6Z 1Y6, Canada; 3 Department of Medicine, University of British Columbia, Vancouver, BC V6Z 1Y6, Canada; 4 Centre for Heart Lung Innovation, St. Paul’s Hospital, Vancouver, BC V6Z 1Y6, Canada

In the original article [1], there was a mistake in Table 1 as published. Reference citation [34] was wrong. The corrected Table 1 appears below. The authors state that the scientific conclusions are unaffected. The original article has been updated.

In the original article [1], there was a mistake in Table 2 as published. Subtitle “Lumacaftor/Ivacaftor” was wrong. The corrected Table 2 appears below. The authors state that the scientific conclusions are unaffected. The original article has been updated.

In the original article [1], there was a mistake in Table A2 as published. Reference citation [48,49,53,54] were wrong. “Talkwalker” was misspelled. The corrected Table A2 appears below. The authors state that the scientific conclusions are unaffected. The original article has been updated.

## Figures and Tables

**Table 1:** Summary of characteristics and results of cohort or survey studies with full manuscript.

Ref	Study Design & Location	Population ^a^	*n*	Recruitment Period & Follow-Up Duration	Overall Adverse Events (AE) ^b,c^			Dose Modification, Interruption, or Discontinuation Due to AE ^b,c^		
Ivacaftor										
[29]	Prospective Cohort ^d^ United States	**Baseline Age** Pediatric and Adult - Mean: 33 yr - Range: 10–61 yr **CFTR Genotype** ≥1 copy G551D **Baseline ppFEV_1_** Mean: 30%	44	**Recruitment Period** Prior to commercial availability **Follow-Up Duration** NS	**AE in *n* = 38 (86%):**	*n*	%	**Discontinuation:**	*n*	%
					- Pulmonary exacerbation - Hemoptysis - Increased sputum - Increased cough - URTI - Dyspnea - Abnormal respiration - Respiratory tract congestion - Headache - Rash	20 7 7 6 6 3 3 3 5 4	45 16 16 14 14 7 7 7 11 9	- Severe abdominal pain - Dizziness/tinnitus	1 1	2 2
					**SAE in *n* = 14 (32%):**					
					- Pulmonary exacerbation - Hemoptysis - Pneumothorax - Acute respiratory failure - URTI - Abdominal pain - Gastroenteritis - Abnormal LFTs - Syncope - Secondary adrenocortical insufficiency	NS NS NS NS NS NS NS NS NS NS	- - - - - - - - - -			
[55]	Prospective Cohort United States Canada Italy	**Baseline Age** Pediatric and Adult - Mean: 17 yr - Range: 5–61 yr **CFTR Genotype** ≥1 gating mutation **Baseline ppFEV_1_** Mean: 86%	23	**Recruitment Period** Mar 2014 to Aug 2015 **Follow-Up Duration** 3 mo	**49 AE in *n* = 21 (91%):**	*n*	%	None reported		
					- Respiratory, unspecified - Gastrointestinal, unspecified - Infection, unspecified - Headache - Weakness - Dizziness - Fatigue	NS NS NS NS NS NS NS	- - - - - - -			
					**5 SAE in *n* = 3 (13%):**	*n*	%			
					- Respiratory infection - Acute changes in metabolic and liver status	4 1	17 4			
[56]	Retrospective Cohort United Kingdom (1 center)	**Baseline Age** Pediatric - Mean: 9 yr - Range: 6–14 yr **CFTR Genotype** 1 copy G551D **Baseline ppFEV_1_** Mean: 68% ^e^	4	**Recruitment Period** Jan 2013 to Jun 2015 **Follow-Up Duration** Mean: 24 mo	- Transaminitis (<3 x ULN)	*n* 1	% 25	None reported		
[57]	Retrospective Cohort Scotland (11 centers)	**Baseline Age** Pediatric - Median: 9 yr **CFTR Genotype** ≥1 copy G551D **Baseline ppFEV_1_** Mean: 85%	26	**Recruitment Period** NS (Jan 2013 to Mar 2013 for 85%) **Follow-Up Duration** Mean: 17 mo		*n*	%	None reported		
					- Headache - Swollen ear - Cataracts	1 1 2	4 4 17 ^f^			
[58]	Retrospective Cohort France (25 centers)	**Baseline Age** Pediatric and Adult - Median: 18 yr - Range: 6–52 yr **CFTR Genotype** ≥1 copy G551D **Baseline ppFEV_1_** Mean: 72%	57	**Recruitment Period** Pre-1 Jun 2013 up to 30 Sep 2014 **Follow-Up Duration** Up to 2 yr	**34 AE in *n* = 21 (37%):**	*n*	%	**Interruption in *n* = 7 (12%):**	*n*	%
					- Transaminitis - Rhinopharyngitis - Asthma - Fever - Chest pain - Abdominal pain - Nausea or vomiting - Intestinal dysmotility - Headache - Fatigue - Rash or eczema - Depression - Myalgia - Arthritis - Breast hypertrophy - Orchitis - Atrial fibrillation	3 NS NS NS NS NS NS NS NS NS NS NS NS NS NS NS NS	5 - - - - - - - - - - - - - - - -	- Hepatitis - Rhinopharyngitis - Abdominal pain - Vomiting - Headache - Rash - Severe depression	NS NS NS NS NS NS NS	- - - - - - -
								**Discontinuation:**		
								- Transaminitis - Liver cirrhosis diagnosis	1 1	2 2
[30]	Retrospective Cohort ^d^ Germany (multicenter)	**Baseline Age** Adult - Mean: 34 yr **CFTR Genotype** ≥1 copy G551D **Baseline ppFEV_1_** Mean: 25%	14	**Recruitment Period** Sep 2012 to Apr 2013 **Follow-Up Duration** Mean: 235 days	- Increased bronchial and nasal secretions - Headache - Worsening RLS - Abdominal pain - Hyperbilirubinemia (mild) - Transaminitis (<3 to 4x ULN)	*n* 3 1 1 1 1 1	% 21 7 7 7 7 7	**Discontinuation:** - Increased bronchial and nasal secretions * * Trial of reduced dose before discontinuation	*n* 1	% 7
**Lumacaftor/Ivacaftor**										
[41]	Prospective Cohort France (1 center)	**Baseline Age** Pediatric - Mean: 16 yr **CFTR Genotype** ∆F508/∆F508 **Baseline ppFEV_1_** Mean: 87%	32	**Recruitment Period** Mar 2016 to Dec 2016 **Follow-Up Duration** 4 h post-first dose	- Acute drop in ppFEV_1_ - Wheeze	*n* 32 3	% 100 9	None reported		
					
[42]	Prospective Cohort France (47 centers)	**Baseline Age** Pediatric and Adult - Mean: 22 yr **CFTR Genotype** ∆F508/∆F508 **Baseline ppFEV_1_** Mean: 65%	845	**Recruitment Period** 1 Jan 2016 to 31 Dec 2016 **Follow-Up Duration** 12 mo	**AE in *n* = 494 (59%):**	*n*	%	**Interruption:**	*n*	%
					- Respiratory - Digestive - Menstrual abnormality - Fatigue - Headache - CK > 5xULN - Transaminitis (> 3xULN)	316 181 53 37 19 20 5	37 21 6 4 2 2 0.6	- Respiratory - ‘Non-respiratory’ **Discontinuation:** Respiratory - Chest tightness/dyspnea - Bronchospasm - Increased cough/sputum - Hemoptysis - Pneumothorax Non-respiratory - Diarrhea, abdominal pain - CK >10xULN + myalgia - Fatigue - Headache - Depression - Metrorrhagia - Transaminitis (>6xULN) - Cutaneous rash - Tachycardia	16 8 *n* 38 24 9 2 1 18 5 5 4 4 3 2 1 1	2 1 % 5 3 1 0.2 0.1 2 0.6 0.6 0.5 0.5 0.4 0.2 0.1 0.1
[59]	Prospective Cohort United States (1 center)	**Baseline Age** Pediatric and Adult - Mean: 23 yr - Range: 12–48 yr **CFTR Genotype** ∆F508/∆F508 **Baseline ppFEV_1_** Mean: 70%	26	**Recruitment Period** NS **Follow-Up Duration** 6 mo	See **Discontinuation**			**Discontinuation:** - Transaminitis - Unspecified AE	*n* 1 4	% 4 15
[31]	Prospective Cohort ^d^ Switzerland (1 center)	**Baseline Age** Adult - Mean NR **CFTR Genotype** ∆F508/∆F508 **Baseline ppFEV_1_** Median: 30%	20	**Recruitment Period** Jan 2016 to Jan 2017 **Follow-Up Duration** 1 mo		*n*	%	**Reduced dose:**	*n*	%
					- Dyspnea - 3 h - 24 h - 1 mo - Chest tightness - 3 h - 24 h - 1 mo - Increased sputum - 3 h - 24 h - 1 mo - Pulmonary exacerbation - 1 mo	0 1 1 1 10 1 1 8 3 2	- 5 5 5 50 5 5 40 15 10	- Respiratory intolerance **Discontinuation:** - Chest tightness (at 24 h)	3 1	15 5
[32]	Prospective Cohort Australia (1 center)	**Baseline Age** Adult - Mean: 27 yr **CFTR Genotype** ∆F508/∆F508 **Baseline ppFEV_1_** Median: 36%	12	**Recruitment Period** Jan 2016 to Oct 2016 **Follow-Up Duration** 1 mo		*n*	%	**Discontinuation:**	*n*	%
					- Acute drop in ppFEV_1_ - Respiratory AE overall - 4 h - 24 h - 1 mo - Dyspnea - 4 h - 24 h - 1 mo - Chest tightness - 4 h - 24 h - 1 mo - Increased sputum - 4 h - 24 h - 1 mo - Pulmonary exacerbation	12 5 10 8 2 6 7 4 8 5 0 2 1 6	100 42 83 67 17 50 58 33 67 42 - 17 8 50	- Chest tightness/dyspnea * * *n* = 2 discontinued after 1mo follow-up (5 wk and 9 wk)	3	25
[33]	Prospective Cohort France (11 centers)	**Baseline Age** Adult - Mean: 31 yr **CFTR Genotype** ∆F508/∆F508 **Baseline ppFEV_1_** Mean: 32%	53	**Recruitment Period** Jan 2016 to Jun 2016 **Follow-Up Duration** 3 mo	**AE in *n* = 34 (63%)**:	*n*	%	**Discontinuation:**	*n*	%
					- Abnormal respiration - Dyspnea - Increased cough - Abdominal pain, nausea, diarrhea, or vomiting - Fatigue - Rash - Pruritus - Breast tension	13 11 3 9 2 1 1 1	25 21 6 17 4 2 2 2	- Respiratory intolerance - Vomiting - Fatigue	13 1 1	25 2 2
[25]	Prospective Cohort ^d,g^ Australia (1 center)	**Baseline Age** Adult - Mean: 27 yr **CFTR Genotype** ∆F508/∆F508 **Baseline ppFEV_1_** Mean: 36%	10	**Recruitment Period** NS **Follow-Up Duration** 52 wk	**AE in *n* = 6 (60%):**	*n*	%	None reported		
					- Chest tightness/dyspnea - Headache	6 2	60 20			
[43]	Retrospective Cohort Ireland (1 center)	**Baseline Age** Pediatric - Mean: 14 yr **CFTR Genotype** ∆F508/∆F508 **Baseline ppFEV_1_** Mean: 77%	15	**Recruitment Period** Sep 2016 to Aug 2017 **Follow-Up Duration** NS		*n*	%	None reported		
					- Acute drop in ppFEV_1_ - Chest tightness - Increased sputum	14 2 2	93 13 13			
[44]	Retrospective Cohort United States (1 center)	**Baseline Age** Pediatric and Adult - Mean: 25 yr - Range: 12–59 yr **CFTR Genotype** ∆F508/∆F508 **Baseline ppFEV_1_** Mean: 67%	116	**Recruitment Period** NS **Follow-Up Duration** Up to 11 mo	**AE in *n* = 46 (40%):**	*n*	%	**Reduced dose:**	*n*	%
					- Chest tightness - Dyspnea - Increased cough - Diarrhea - Nausea - Decreased appetite - Rash	23 12 10 5 3 2 2	20 10 9 4 3 2 2	- AE not specified **Discontinuation:** - Reasons not specified ^h^	10 20	9 17
[60]	Retrospective Cohort Greece (1 center)	**Baseline Age** Pediatric and Adult - Mean: 16 yr ^i^ - Range: 12–23 yr **CFTR Genotype** ∆F508/∆F508 **Baseline ppFEV_1_** Mean 92% ^i^	62	**Recruitment Period** Mar 2016 to Aug 2017 **Follow-Up Duration** 12 mo	- Chest tightness	*n* 2	% 3	**Discontinuation:** - Transaminitis - Cataract	*n* 1 1	% 2 2
[34]	Retrospective Cohort ^d^ Spain (multicenter)	**Baseline Age** Pediatric and Adult - Mean: 27 yr - Range: 10–45 yr **CFTR Genotype** ∆F508/∆F508 **Baseline ppFEV_1_** Mean: 32%	20	**Recruitment Period** 2016 **Follow-Up Duration** 6 mo	**AE in *n* = 15 (75%):**	*n*	%	**Discontinuation:**	*n*	%
					- Chest tightness - Dyspnea - Headache - Weight loss - ‘Sickness’ (not defined) - Asthenia - Abdominal pain - Transaminitis	9 8 5 5 3 3 2 2	45 40 25 25 15 15 10 10	- Decreased ppFEV_1_ - AE not specified	1 6	5 30
[45]	Retrospective Cohort Canada (1 center)	**Baseline Age** Adult - Median: 32 yr **CFTR Genotype** ∆F508/∆F508 **Baseline ppFEV_1_** Median: 40%	22	**Recruitment Period** Apr 2016 to Jun 2018 **Follow-Up Duration** Median: 10 mo	**AE in *n* = 19 (86%):**	*n*	%	**Discontinuation:**	*n*	%
					- Chest tightness - Wheeze - Dyspnea - Increased sputum - Increased cough - Flu-like symptoms - Elevated blood pressure - Headache - Nausea - Elevated AST - Anxiety - Bradycardia - Pleuritic chest pain	14 4 3 3 2 1 5 4 2 1 1 1 1	64 18 14 14 9 5 23 18 9 5 5 5 5	- Respiratory symptoms - Asymptomatic hypertension - Symptomatic hypertension - Headache - Hypertensive emergency - Anxiety	3 2 1 1 1	14 9 5 5 5
[61]	Retrospective Cohort United States (1 center)	**Baseline Age** Adult - Mean NR **CFTR Genotype** ∆F508/∆F508 **Baseline ppFEV_1_** Mean NR	82	**Recruitment Period** Jul 2015 to Jun 2016 **Follow-Up Duration** 12 mo	See **Discontinuation**			**Discontinuation:** Total overall: - Chest tightness * - Diarrhea ** - Abdominal pain - Nausea ** - Dysphagia - Elevated LFTs - Pericarditis - Allergic reaction ** - Suspected Stevens–Johnson syndrome * *n* = 3 also had significant drop in ppFEV_1_ ** *n* = 1 also discontinued due to chest tightness	*n* 17 11 2 1 1 1 1 1 1 1	% 21 13 2 1 1 1 1 1 1 1
[26]	Retrospective Cohort ^d,g^ Australia (7 centers)	**Baseline Age** Adult - Mean: 31 yr **CFTR Genotype** ∆F508/∆F508 **Baseline ppFEV_1_** Mean: 37%	72	**Recruitment Period** Nov 2015 to Mar 2017 **Follow-Up Duration** 12 mo		*n*	%	**Discontinuation:**	*n*	%
					- Chest tightness/dyspnea - Increased sputum - Decrease in ppFEV_1_ - Headache - Fatigue - Nausea - Rash	40 4 2 2 5 1 2	56 6 3 3 7 1 3	- Chest tightness/dyspnea	22	31
[27]	Case Series (Survey) ^j^ International (31 centers)	**Baseline Age** Adult - Mean: 30 yr **CFTR Genotype** ∆F508/∆F508 **Baseline ppFEV_1_** Mean: 59%	26	**Recruitment Period** Questionnaire sent in 2018–2019 **Follow-Up Duration** NS		*n*	%	**Discontinuation:**	*n*	%
					- Pulmonary exacerbation - Post-partum acute myelocytic leukemia	1 1	4 4	- Chest tightness	2	8

^a^ When adult and pediatric patients both included, age range reported when possible; ^b^ Rates not reported for all AE, as indicated by ‘NS’; ^c^ To avoid redundancy, if AE only reported in context of dose modification, interruption, and/or discontinuation of therapy, it was not listed in overall AE; ^d^ Study population part of a compassionate, ‘expanded access’, ‘managed access’, or ‘named patient’ program; ^e^ Mean calculated from *n* = 3 (75%) of study subjects, as baseline not reported for *n* = 1 (25%); ^f^ Frequency of 17% based on *n* = 12 screened; 8% frequency for overall cohort of *n* = 26; ^g^ Study was case-control, but only LUM/IVA-treated participants included in systematic review; therefore, assessed as cohort study; ^h^ Reason for discontinuation was not consistently assessed, and may include reasons unrelated to AE; ^i^ Mean baseline age and ppFEV_1_ based on *n* = 52 in final analysis of outcomes assessing effectiveness; *n* = 10 excluded from this analysis; ^j^ This case series is included in Table 1 due to results being presented in aggregate. **AST**, aspartate aminotransferase; **CFTR**, cystic fibrosis transmembrane conductance regulator; **CK**, creatine kinase; **h**, hour(s); **LFT**, liver function test; **mo**, month(s); **NR**, not reported; **NS**, not specified; **ppFEV_1_**, percent predicted Forced Expiratory Volume in 1 sec; **RLS**, restless leg syndrome; **SAE**, serious adverse events; **ULN**, upper limit of normal; **URTI**, upper respiratory tract infection; **wk**, week(s); **yr**, year(s).

**Table 2:** Summary of characteristics and results of cohort or survey studies in abstract form.

Ref	Study Design	Population	*n*	Overall Adverse Events (AE) ^a,b^			Dose Modification, Interruption, or Discontinuation Due to AE ^a,b^		
Ivacaftor									
[62]	Prospective Cohort	**Baseline Age** Pediatric - Mean: 5 yr **CFTR Genotype** ≥1 gating mutation **Baseline ppFEV_1_** Mean NR	4	**AE in *n* = 2 (50%):**	*n*	%	**None reported**		
				- URTI - Nasal congestion - Headache	NS NS NS	- - -			
[63]	Retrospective Cohort	**Baseline Age** Pediatric - Mean: 6 yr **CFTR Genotype** ≥1 gating mutation **Baseline ppFEV_1_** Median: 87%	10	- Transient rash - Increased obesity	*n* 2 1	% 20 10	None reported		
[64]	Prospective Cohort	**Baseline Age** Pediatric and Adult - Mean NR **CFTR Genotype** ≥1 copy S549R **Baseline ppFEV_1_** Mean: 54%	15	- Liver enzyme derangement	*n*	%	None reported		
					2	13			
[65]	Cross-sectional Survey	**Baseline Age** Adult - Mean: 26 yr **CFTR Genotype** ≥1G551D **Baseline ppFEV_1_** Mean: 62%	11 ^d^	**AE in *n* = 8 (73%) ^d^:** - Transient rash - Dizziness - Unspecified AE	*n* NS NS NS	% - - -	None reported		
**Lumacaftor/Ivacaftor**									
[66]	Prospective Cohort	**Baseline Age** Pediatric - Mean: 13 yr **CFTR Genotype** ∆F508/∆F508 **Baseline ppFEV_1_** Mean: 91%	14		*n*	%	Reduced dose *:	*n*	%
				- Acute drop in ppFEV_1_ (asymptomatic) - Chest tightness, tachypnea (requiring oxygen)	1 1	7 7	- Chest tightness, tachypnea * Eventual titration to full dose	1	7
[67]	Prospective Cohort	**Baseline Age** Pediatric - Mean: 14 yr **CFTR Genotype** ∆F508/∆F508 **Baseline ppFEV_1_** Mean: 87%	13		*n*	%	None reported		
				- Drop in ppFEV_1_ requiring salbutamol	7	54			
	
[68]	Prospective Cohort	**Baseline Age** Pediatric and Adult - Mean: 23 yr ^e^ **CFTR Genotype** ∆F508/∆F508 **Baseline ppFEV_1_** Mean 61% ^e^	369		*n*	%	**Discontinuation**:	*n*	%
				- Bronchospasm - Dyspnea - Abnormal respiration - Unspecified respiratory AE - Unspecified AE	15 12 7 4 120	4 3 2 1 33	- Unspecified AE	16	4
[69]	Prospective Cohort	**Baseline Age** Pediatric and Adult - Mean: 25 yr **CFTR Genotype** ∆F508/∆F508 **Baseline ppFEV_1_** Mean NR	311	**379 AE in *n* = 213 (68%):** - Dyspnea - Cough - GI discomfort (e.g., diarrhea, nausea, abdominal pain) - Headache - Fatigue - Unspecified	n ^f^ NS NS NS NS NS NS	% 31 6 31 6 5 NR	**Interruption (stop/restart)**: - Unspecified AE and other reasons ^g^ **Discontinuation**: - Unspecified AE and other reasons ^g^	*n* 12 42	% 4 14
[35]	Prospective Cohort ^c^	**Baseline Age** Adult - Median: 31 yr **CFTR Genotype** ∆F508/∆F508 **Baseline ppFEV_1_** Median: 28%	14		*n*	%	**Discontinuation**:	*n*	%
				- Chest tightness, breathless - Rash	7 1	50 7	- Respiratory AE and/or rash	4	29
[70]	Prospective Cohort	**Baseline Age** Adult - Mean NR **CFTR Genotype** ∆F508/∆F508 **Baseline ppFEV_1_** Mean NR	29		*n*	%	**Reduced dose**:	*n*	%
				- Chest tightness * * *n* = 4 cases severe, requiring hospitalization for IV steroids and antibiotics	13	45	- Chest tightness **Discontinuation**: - Chest tightness	2 5	7 17
[36]	Prospective Cohort ^c^	**Baseline Age** Mean NR ^h^ **CFTR Genotype** ∆F508/∆F508 **Baseline ppFEV_1_** Mean NR	32	**AE in 88%**:	n ^f^	%	**Interruption (stop/restart)**:	*n*	%
				- Respiratory AE - Drop in ppFEV_1_	NS NS	87 -	- Unspecified AEA **Discontinuation**: - Unspecified AE	1 8	3 25
[71]	Retrospective Cohort	**Baseline Age** Pediatric and Adult - Mean NR **CFTR Genotype** ∆F508/∆F508 **Baseline ppFEV_1_** Mean NR	34	**AE in *n* = 29 (85%):** - Pulmonary exacerbation - Chest tightness - Dyspnea - Diarrhea - Abdominal pain	*n* 16 9 3 3 3	% 47 26 9 9 9	**Discontinuation**: - Unspecified AE	*n* 10	% 29
				**Serious AE in *n* = 8 (24%)**:					
				- Respiratory failure ^i^ - Unspecified AE	1 7	3 21			
[72]	Retrospective Cohort	**Baseline Age** Pediatric and Adult - Mean: 26 yr **CFTR Genotype** ∆F508/∆F508 **Baseline ppFEV_1_** Mean: 68%	103	See **Discontinuation**			**Interruption/discontinuation ^j^**:	*n*	%
							- Chest tightness and/or pain - Elevated LFTs	17 NS	17 -
[73]	Retrospective Cohort	**Baseline Age** Adult - Mean: 31 yr **CFTR Genotype** ∆F508/∆F508 **Baseline ppFEV_1_** Mean: 50%	71	**AE in *n* = 41 (58%):**	*n*	%	**Discontinuation**:	*n*	%
				- Chest tightness - Dyspnea - Increased cough - GI (pain, constipation, or diarrhea) - Rash - Pruritus - Irregular menses or metrorrhagia - Breast tension - Headache - Myalgia	22 8 4 6 4 1 3 2 1 1	31 11 6 9 6 1 4 3 1 1	- Dyspnea - Chest tightness - Increased cough - Fatigue	7 6 3 1	10 9 4 1
[74]	Retrospective Cohort	**Baseline Age** Mean NR ^h^ **CFTR Genotype** ∆F508/∆F508 **Baseline ppFEV_1_** Mean NR	54	**See Discontinuation**			**Discontinuation**:	*n*	%
							- Chest tightness, dyspnea, and/or drop in ppFEV_1_	8	15
[75]	Retrospective Cohort	**Baseline Age** Adult - Mean: 31 yr **CFTR Genotype** ∆F508/∆F508 **Baseline ppFEV_1_** Mean NR	28	- Increased work of breathing or chest tightness - Drop in ppFEV_1_	*n* 12 5	% 43 18	**Discontinuation** - Respiratory intolerance vs. pulmonary exacerbation - Persistent respiratory intolerance/chest tightness - Rash and swelling of face - Increased anxiety	*n* 1 3 1 1	% 4 11 4 4
[76]	Retrospective Cohort	**Baseline Age** Adult - Mean NR **CFTR Genotype** ∆F508/∆F508 **Baseline ppFEV_1_** Mean NR	46		*n*	%	**Discontinuation**:	*n*	%
				- Drop in ppFEV_1_ - Transaminitis	21 2	46 4	- Dyspnea, cough, CFPEx, and/or chest tightness - Transaminitis - Headache - Muscle ache - Fatigue - Rash	4 1 NS NS NS NS	9 2 - - - -
[77]	Retrospective Cohort	**Baseline Age** Adult - Mean NR **CFTR Genotype** ∆F508/∆F508 **Baseline ppFEV_1_** Mean NR	28	See **Discontinuation**			**Discontinuation**:	*n*	%
							- SOB and/or drop in ppFEV_1_	15	54
[78]	Retrospective Cohort	**Baseline Age** Mean: 32 yr ^h^ **CFTR Genotype** ∆F508/∆F508 **Baseline ppFEV_1_** Mean: 62%	20	**Overall AE**:	*n*	%	**Interruption (stop/restart):**	*n*	%
				- Chest tightness - Elevated LFTs - Upset stomach - Increased stool output - Rash - Elevated thyroid function test - RA exacerbation	NS NS NS NS NS NS NS	- - - - - - -	- Unspecified AE - full-dose restart - half-dose restart **Discontinuation**: - Unspecified AE	2 4 2	10 20 10
[79]	Retrospective Cohort	**Baseline Age** Mean NR ^h^ **CFTR Genotype** ∆F508/∆F508 **Baseline ppFEV_1_** Mean NR	60	- Heartburn/reflux - Abdominal pain - Loose/oily stools	*n* 20 19 17	% 33 32 28	None reported		
[80]	Retrospective Cohort	**Baseline Age** Mean: 29 yr ^h,k^ **CFTR Genotype** ∆F508/∆F508 **Baseline ppFEV_1_** Mean: 80% ^k^	34	See **Discontinuation**			**Discontinuation**:	*n*	%
							Overall total: - Respiratory AE (70%) ^f^ - Unspecified reasons ^g^	11 NS NS	32 - -
[81]	Cohort ^l^	**Baseline Age** Pediatric - Mean NR **CFTR Genotype** ∆F508/∆F508 **Baseline ppFEV_1_** Mean NR	39		*n*	%	None reported		
				- AST >3x ULN	2	5			
[82]	Cohort ^l^	**Baseline Age** Pediatric and Adult - Range: 13–48 yr **CFTR Genotype** ∆F508/∆F508 **Baseline ppFEV_1_** Mean NR	47	**See Discontinuation**			**Discontinuation**:	*n*	%
							- Thoracic oppression and unspecified AE	4	9
[83]	Cohort ^l^	**Baseline Age** Adult - Mean: 28 yr **CFTR Genotype** ∆F508/∆F508 **Baseline ppFEV_1_** Mean: 61%	46	See **Discontinuation**			**Discontinuation**:	*n*	%
							- Dyspnea, increased sputum, and unspecified AE	6	13
[37]	Cohort ^c,l^	**Baseline Age** Adult - Mean: 31 yr **CFTR Genotype** ∆F508/∆F508 **Baseline ppFEV_1_** Mean: 28%	30	- Drop in ppFEV_1_ - Dyspnea, chest tightness, or chest pain - Increased sputum *Based on 31 trials of LUM/IVA in 30 patients	*n* 30 * 25 * NS	% 97 81 -	**Discontinuation**: - Respiratory AE, unspecified - Hypertension	*n* 3 1	% 10 3
[84]	Cohort ^l^	**Baseline Age** Adult - Mean: 31yr **CFTR Genotype** ∆F508/∆F508 **Baseline ppFEV_1_** Mean: 40%	8	See **Interruption**			**Interruption in *n* = 1 (13%)**:	*n*	%
							- Drop in ppFEV_1_ - Eczema	1 1	13 13
[38]	Cohort ^c,l^	**Baseline Age** Mean NR ^h^ **CFTR Genotype** ∆F508/∆F508 **Baseline ppFEV_1_** Mean NR	19	See **Discontinuation**			**Discontinuation**:	*n*	%
							- Chest tightness and dyspnea	4	21
[85]	Cross-sectional questionnaire	**Baseline Age** Mean NR ^h^ **CFTR Genotype** ∆F508/∆F508 **Baseline ppFEV_1_** Mean NR	11	**AE in *n* = 5 (46%)**: - Increased cough - Chest pain - Trouble breathing - Chest tightness - Stomach pain	*n*	%	**Discontinuation**:	*n*	%
					4 2 2 1 1	36 18 18 9 9	- Increased cough	1	9
**Tezacaftor/Ivacaftor**									
[86]	Prospective Cohort	**Baseline Age** Pediatric - Mean: 16 yr **CFTR Genotype** ∆F508 homozygous or heterozygous **Baseline ppFEV_1_** Mean: 82%	72	See **Discontinuation**			**Discontinuation**: Overall total: - New-onset hemoptysis - Persistent nausea/vomiting - Elevated LFTs - Mental health changes - Alterations in blood glucose - Acholic stools	*n* 8 NS NS NS NS NS NS	% 11 - - - - - -
[87]	Prospective Cohort	**Baseline Age** Mean NR ^h^ **CFTR Genotype** NR **Baseline ppFEV_1_** Mean NR	50		*n*	%	**Discontinuation**:	*n*	%
				- AE not specified	5	10	- Liver function abnormalities	1	2
[88]	Prospective Cohort	**Baseline Age** Adult - Mean: 34 yr **CFTR Genotype** ∆F508/∆F508 **Baseline ppFEV_1_** Mean: 51%	5 ^m^	**AE in *n* = 5 (11%) ^m^**:	*n*	% ^m^	**Discontinuation**:	*n*	% ^m^
				- Sleep pattern disturbance - Out of body experience - Visual hallucination - Depersonalization - “Brain fog” - Severe migraine	2 1 1 1 1 1	5 2 2 2 2 2	- Out of body experience, visual hallucination - Depersonalization, “brain fog” - Severe migraine	1 1 1	2 2 2
[89]	Retrospective Cohort	**Baseline Age** Adult - Mean NR **CFTR Genotype** ∆F508/∆F508 **Baseline ppFEV_1_** Mean NR	18	See **Discontinuation**			**Discontinuation**:	*n*	%
							- Hair loss and fatigue	1	6
[39]	Cohort ^c,l^	**Baseline Age** Adult - Mean NR **CFTR Genotype** NR **Baseline ppFEV_1_** Mean: 34%	22	**AE in *n* = 3 (14%)**: - Rash - Blurred vision - Viral symptoms	*n*	%	**Discontinued**:	*n*	%
					2 1 1	9 5 5	- Blurred vision	1	5
**Elexacaftor/Tezacaftor/Ivacaftor**									
[40]	Retrospective Cohort	**Baseline Age** Adult - Mean: 36 yr **CFTR Genotype** ≥1 copy ∆F508 **Baseline ppFEV_1_** Mean: 31%	11		*n*	%	None reported		
				- Transaminitis	4	36			

^a^ Rates not reported for all AE, as indicated by ‘NS’; ^b^ To avoid redundancy, if AE only reported in context of dose modification, interruption, and/or discontinuation of therapy, it was not listed in overall AE; ^c^ Study population part of a compassionate, ‘early access’, ‘expanded access’, ‘managed access’, or ‘named patient’ program; ^d^ Total study cohort of *n* = 11, but only *n* = 9 patients completed symptom questionnaire; AE frequency calculated based on total *n* = 11, ^e^ Mean baseline age and ppFEV_1_ based on *n* = 135 in final analysis of outcomes assessing effectiveness; *n* = 234 excluded from this analysis; ^f^ As reported, unable to accurately determine the absolute number of patients who experienced AE; ^g^ Frequency of specific reasons for interruption or discontinuation not clear and include reasons unrelated to AE; ^h^ Included age groups (i.e., pediatric and/or adult) not specified; ^i^ Respiratory failure occurred in 1 individual on two occasions, both within 24 h of initiating and reinitiating LUM/IVA; ^j^ Of the *n* = 25 who stopped, 9 restarted and 6 of experienced the same AE; unclear which AE the 3 who restarted experienced and whether the 6 who experienced the same AE then discontinued permanently; ^k^ Mean baseline age and ppFEV_1_ based on *n* = 23 in final analysis of outcomes assessing effectiveness; *n* = 11 excluded from this analysis; ^l^ Unable to discern if prospective versus retrospective based on reported information; ^m^ Total study cohort of *n* = 44, but focused on neurocognitive AE in *n* = 5; AE frequencies calculated based on total *n* = 44. **AST**, aspartate aminotransferase; **CFTR**, cystic fibrosis transmembrane conductance regulator; **CFPEx**, cystic fibrosis pulmonary exacerbation; **GI**, gastrointestinal; **LFT**, liver function test; **LUM/IVA**, lumacaftor/ivacaftor; **NR**, not reported; **NS**, not specified; **ppFEV_1_,** percent predicted forced expiratory volume in 1 sec; **RA**, rheumatoid arthritis; **SOB**, shortness of breath; **ULN**, upper limit of normal; **URTI**, upper respiratory tract infection; **yr,** year(s).

**Table A2:** Summary of methodological ratings of included case series ^a,b.^

Criteria	McKinzie et al., 2017 [47]	Nash et al., 2020 [27]	Rotolo et al., 2020 [52]	Safirstein et al., 2020 [53]	Talwalkar et al., 2017 [48]
1. Study objective clearly stated	Y	Y	N	Y	Y
2. Study population clearly defined, using case definition	N	N	N	N	N
3. Cases consecutive	NR	NR	NR	NR	NR
4. Subjects comparable	CD	CD	CD	N	N
5. Intervention clearly described	Y	Y	Y	Y	Y
6. Outcome measures clearly defined, valid, reliable, implemented consistently	N	N	N	Y	N
7. Adequate length of follow-up	Y	Y	Y	Y	CD
8. Statistical methods well-described	N/A	N/A	N/A	N/A	N
9. Results well-described	Y	N	Y	Y	Y
**Final rating**	**Poor**	**Poor**	**Fair**	**Good**	**Poor**

^a^ Studies were rated against the 9 criteria of the Quality Assessment for Case Series Studies from the National Institutes of Health, National Heart, Lung, and Blood Institute [24] from the standpoint of AE assessment; ^b^ Only case series with a full manuscript were assessed for quality. **AE**, adverse event; **CD**, cannot determine; **N**, no; **N/A**, not applicable; **NR**, not reported; **Y**, yes.
